# Rapid regeneration offsets losses from warming-induced tree mortality in an aspen-dominated broad-leaved forest in northern China

**DOI:** 10.1371/journal.pone.0195630

**Published:** 2018-04-06

**Authors:** Pengwu Zhao, Chongyang Xu, Mei Zhou, Bo Zhang, Peng Ge, Nan Zeng, Hongyan Liu

**Affiliations:** 1 Academy of Forestry, Inner Mongolia Agricultural University, Huhhot, Inner Mongolia, China; 2 College of Urban and Environmental Sciences and MOE Laboratory for Earth Surface Processes, Peking University, Beijing, China; Chinese Academy of Forestry, CHINA

## Abstract

Worldwide tree mortality as induced by climate change presents a challenge to forest managers. To successfully manage vulnerable forests requires the capacity of regeneration to compensate for losses from tree mortality. We observed rapid regeneration and the growth release of young trees after warming-induced mortality in a David aspen-dominated (*Populus davidiana*) broad-leaved forest in Inner Mongolia, China, as based on individual tree measurements taken in 2012 and 2015 from a 6-ha permanent plot. Warming and drought stress killed large trees 10–15 m tall with a total number of 2881 trees during 2011–2012, and also thinned the upper crowns. David aspen recruitment increased 2 times during 2012–2015 and resulted in a high transition probability of David aspen replacing the same or other species, whereas the recruitment of Mongolian oak (*Quercus mongolica*) was much lower: it decreased from 2012 to 2015, indicating that rapid regeneration represented a regrowth phase for David aspen, and not succession to Mongolian oak. Further, we found that the recruitment density increased with canopy openness, thus implying that warming-induced mortality enhanced regeneration. Our results suggest that David aspen has a high regrowth ability to offset individual losses from warming-induced mortality. This important insight has implications for managing this vulnerable forest in the semi-arid region of northern China.

## Introduction

Tree mortality has been reported in semi-arid regions worldwide [1]. Previous studies have primarily concentrated on the drivers of tree mortality and have linked it to climate change, pest outbreaks, and fire regimes [2–4]. Recently, however, tree regeneration following episodes of mortality—knowledge that is necessary for predicting forest dynamics under future climate change—has become a topic of interest [5].

Among the forest types suffering dieback, trembling aspen (*Populus tremuloides*) ranks among the most biologically diverse vegetation communities across the Intermountain Region of western North America, and this type has been well-studied [5–9]. Trembling aspen mortality and overstory dieback have occurred at unusually high levels throughout Colorado and western North America over the past 15 years [7]. Findings from southwestern Colorado indicated that dieback and mortality were clearly related to climate stress, coupled to disease and insect attacks [10]. It was also found that trembling aspen stands continue to deteriorate after drought-incited sudden aspen decline [5].

Although aspen mortality has been widely documented in North America and Inner Asia [7, 11], studies about aspen regeneration after warming-induced mortality were few. Forest regeneration is commonly associated with various biotic features of tree species, such as the vegetative sprouting of aspen [12, 13]. Interspecific competition usually controls aspen regeneration during the process of secondary succession [8]. Disturbance such as fire is known to have positive effect on aspen regeneration [14–16]. Similarly, heavy partial or even clear-cutting have been suggested in forest management to promote aspen recruitment [17, 18]. In these prior studies, however, tree mortality and regeneration were often studied separately. Recent research indicates that the density of tree seedlings increases in healthy plots but decreases in drought-stricken plots, implying a possible negative relationship between warming-induced mortality and regeneration [5].

In North America, the poor regeneration potential of trembling aspen has been observed in stands affected by severe drought, which might lead to conifer succession or conversion to shrub or grass vegetation, raising concerns that increasing aridity could ultimately lead to the widespread loss of aspen forest cover [7, 8, 19–23]. Although warming-induced mortality of David aspen has been **r**eported in the temperate forest-steppe ecotone of northern China [11, 24], little is known about David aspen regeneration or the succession of mortality in this region. Hence, how to manage those stands that are susceptible to David aspen mortality remains a challenge. Since the regeneration ability of aspen may determine the process and direction of succession, we hypothesized that the ineffective regeneration of David aspen should lead to an alternative successional pathway or forest decline after forest dieback in this region.

To test this hypothesis, we selected a David aspen-dominated forest containing Mongolian oak, white birch (*Betula platyphylla*), and black birch (*Betula dahurica*), in a mountainous region of the forest-steppe ecotone in Inner Mongolia, China. The objectives of this study were to: (1) characterize the regeneration status of David aspen after warming-induced mortality, (2) analyze the changes of the dominant species after tree mortality, and (3) evaluate the relationship between tree mortality and regeneration. Through these, we sought a scientific basis for managing this vulnerable forest, as well as those facing similar threats in other parts of the world.

## Study area and methods

### Site and field monitoring

**Fig 1:**
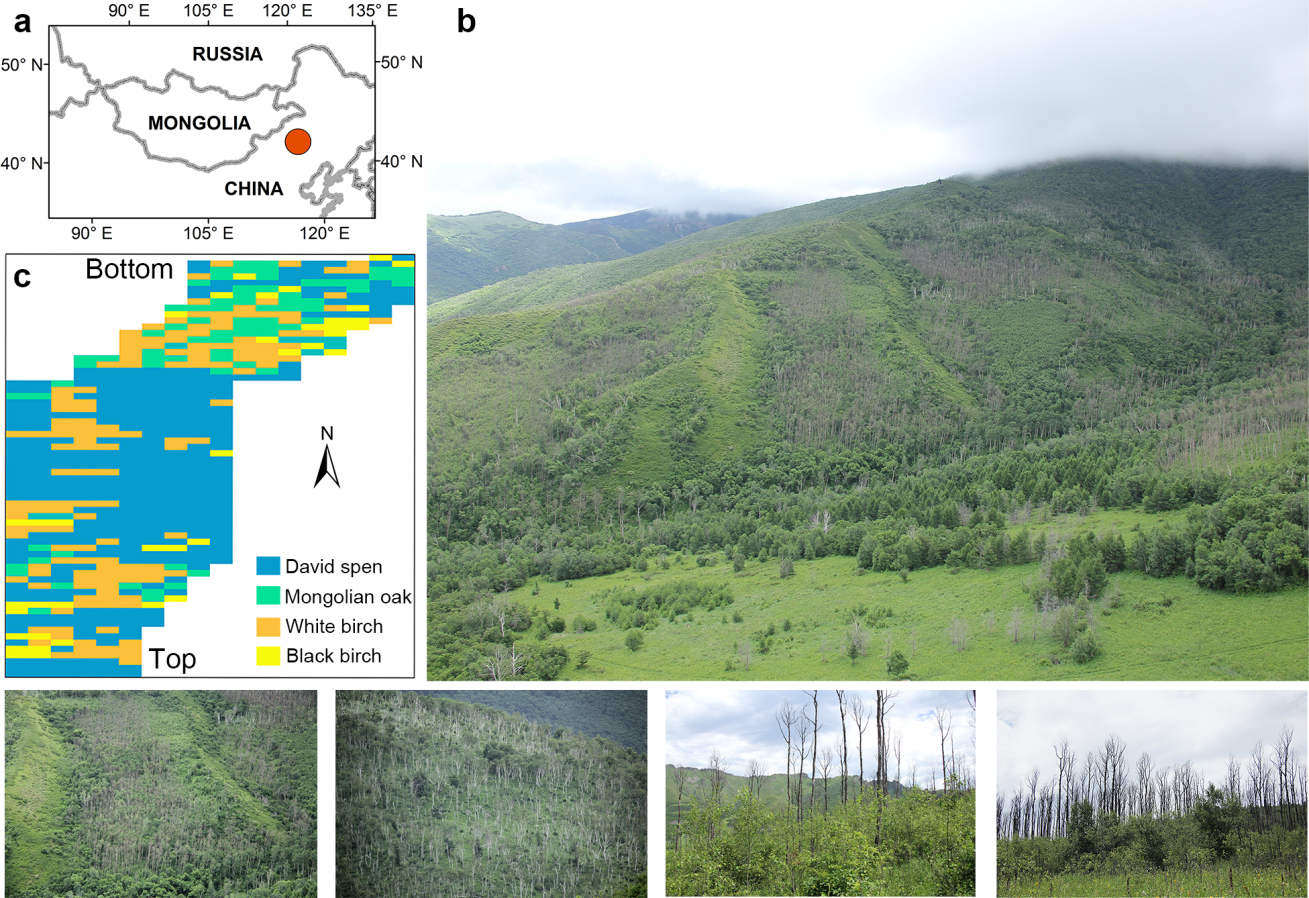
Location of study area and site characteristics. (a) Location of the study area (red dots), (b) landscape overview of the study area, and (c) sketch of the site with spatial distribution of the dominant tree species. The software ArcView 3.2 (ESRI, Redlands, CA, USA, http://www.esri.com) with state border was used to create the map of a). The sketch of the site was drawn based on the field data collected by the authors. The lower photos show examples of tree mortality (two at left) and abundant recruitment around dead trees (two at right) in the study region (taken in August 2014 by Chongyang Xu).

Our study site was in a mountainous region of the forest-steppe ecotone in northern China. In this region, we chose a broad-leaved forest dominated by David aspen and accompanied by Mongolian oak, white birch, and black birch for our investigation (Fig 1). This site is at an elevation of 1175–1355 m a.s.l.; it has annual temperature of c. 2°C and the mean annual precipitation is c. 360 mm [24]. Warming-induced mortality in this region was reported for 2011–2012, with the highest mortality occurring in both aspen and Mongolian oak (mortality ratio > 60%) [24].

In 2012, a permanent plot (6 ha) was established in broad-leaved forest that consisted of 600 subplots, each 10 m × 10 m (100 m^2^), at Saihan Wula National Nature Reserve by Inner Mongolia Agricultural University. Data collection was permitted by the Saihan Wula National Nature Reserve. All individuals with a stem diameter at the base (about 5 cm from ground) > 1 cm in the permanent plot were labeled in 2012. Each individual was assigned a unique number. We identified the species and status (dead or living) of each labeled individual. We also measured the diameter of breast height (DBH) and height of all labeled individuals [24, 25]. The crown of every labeled individual was considered as an ellipse projected onto the ground, and both the lengths of long and short axes were measured to estimate tree crown size (m^2^). All of this 2012 fieldwork was likewise repeated in 2015. Any newly-recruited individuals generated after 2012 were labeled and their DBH and height were measured in 2015.

### Assessment of tree mortality, growth, and regeneration

This study used 600 subplots in 2012 and in 2015 to evaluate the dead tree density, recruitment density, and canopy openness. For each subplot, we calculated the dead tree densities of four species (David aspen, Mongolian oak, white birch, black birch), expressed as the number of dead trees per 100 m^2^. The growth increment of each tree was evaluated by the changes in both DBH and height during the 2012–2015 period; the growth of each of the four species was averaged from co-occurring trees in each subplot. Recruitment in 2015 was defined as the number of new trees per 100 m^2^ that were initiated previously during 2012–2015. These new recruits had ranges in DBH and height of <7 cm and <8 m, respectively. To evaluate the regeneration status before mortality occurred, recruits in 2012 were considered as those young trees with DBH <7 cm and height <8 m. Recruitment densities of the four tree species in each subplot were also assessed as counts of recruitments per 100 m^2^.

The diameter distribution of living trees in 2012 and 2015 were compared to assess changes in the dominant species in the mixed broad-leaved forest after severe mortality. For each of the dominant species, a diameter distribution was constructed by summing the number of living trees in 5-cm DBH intervals, and then dividing by the total number of living trees to yield a relative frequency (%). We further build up a transition probability matrix over the period of 2012 to 2015 to illustrate transition dynamics between the four tree species after tree mortalities [26, 27]. The subplots with mortality > 0 were considered as gaps which were released by tree death and available for capture. The transition probabilities of four tree species replaced by another of the same or a different species were estimated by counting the dominant tree species in 2012 and the percentage of seedlings and saplings of the various species.

We used the density of the surviving trees and canopy openness to estimate within-community competition after tree mortality, since competition among living trees predominates in the forest communities of this study region [28–30]. The number of surviving trees was counted within each subplot. The canopy openness in each subplot was considered as ratio of gap areas accounting for the total area of each subplot. In previous studies, canopy openness was usually measured with hemispherical photographs or with equipment measuring photosynthetic active radiation [29–31]. However, both methods cannot reflect the openness changes caused by tree mortality and recruitment. In our study, crown of each tree was considered as an ellipse, and the location of each tree was considered as the ellipse’s center. Ellipse of every tree in each subplot was projected on the same horizontal plane to roughly reconstruct the horizontal structure of canopy. Canopy openness was estimated by the total area without tree’s crown divided by the area of each subplot. As coordinates of each tree in each subplot were recorded in the field, the overlapped area can be excluded in calculation. As we have recorded coordinates of each tree and lengths of long and short axes of each tree crown, it is quite practical to use our method to estimate the openness of each subplot for a 6-ha permanent plot to study the relationship between openness changes and tree mortality/recruitment.

Canopy openness was estimated by the total area without tree’s crown divided by the area of each subplot. Linear regression was used to evaluate the relationship between recruitment density and (1) canopy openness and (2) surviving tree density.

All the raw data can be found in the supporting information S1 Table.

## Results

### Increased tree growth and regeneration during 2012–2015

The tree stem DBH and the height of the four dominant species increased during 2012–2015, but especially for David aspen, for which DBH increased by 34.9% and height increased by 18.0%. The DBH increment of Mongolian oak was 18.9%, and its height increment was 12.2%. For both white birch and black birch, their DBH increased by 11%, and their height increased by 8%. Increments of DBH and height for David aspen and Mongolian oak mainly occured in relatively young trees in the 0–10 cm diameter class, which had 1.5–2.5 cm increments for DBH (S1A Fig, see Table A in S1 Table) and 0.5–1.5 m increments for height (S1B Fig, see Table B in S1 Table). By contrast, for white birch and black birch, their increments of DBH occurred in trees over a relatively wide range (0–20 cm) of diameter classes (S1 Fig).

**Fig 2:**
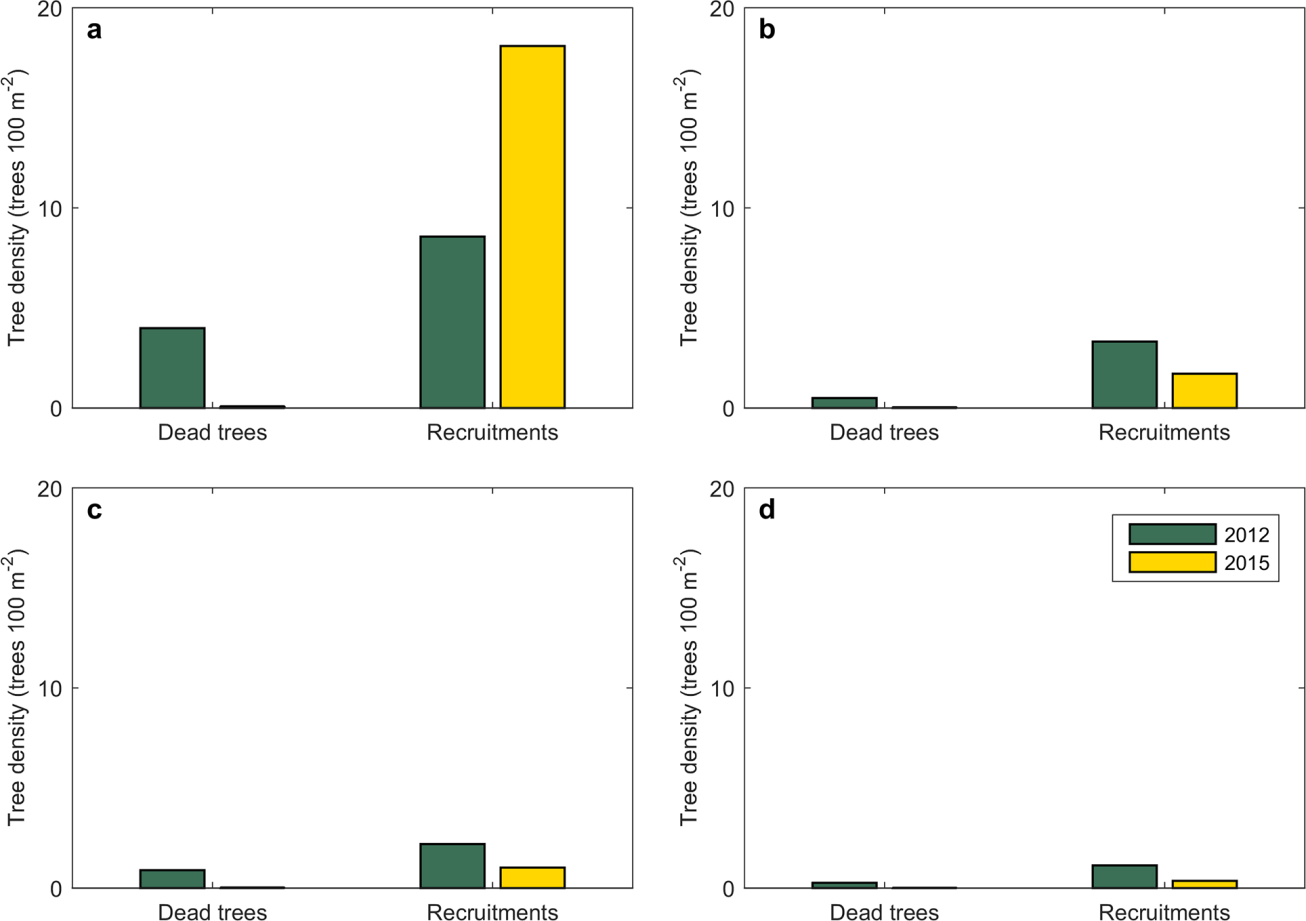
The density of dead trees and the recruitment of four tree species. (a) David aspen, (b) Mongolian oak, (c) white birch, and (d) black birch in 2012 (green) and 2015 (orange).

In 2012, the density of dead David aspen was 4.0 trees 100 m^−2^ (Fig 2A), followed by the density of dead white birch (0.8 trees 100 m^−2^, Fig 2C, see Table C in S1 Table). Comparatively few trees died during 2012–2015. The recruitment density of David aspen in 2015 doubled that in 2012 (18.1 trees 100 m^−2^ vs. 8.5 trees 100 m^−2^; Fig 2A, see Table D in S1 Table). The recruitment densities of the other three species were clearly lower than that of David aspen, and the recruitment densities of Mongolian oak, white birch, and black birch in 2015 were relatively lower than the corresponding values in 2012 (Fig 2B–2D). For example, the recruitment density for Mongolian oak in 2012 was 3.3 trees 100 m^−2^, whereas in 2015 it was 1.7 trees 100 m^−2^ (Fig 2B). The total number of living trees increased from 13815 in 2012 to 18436 in 2015.

### Change in the diameter distributions of tree species during 2012–2015

**Fig 3:**
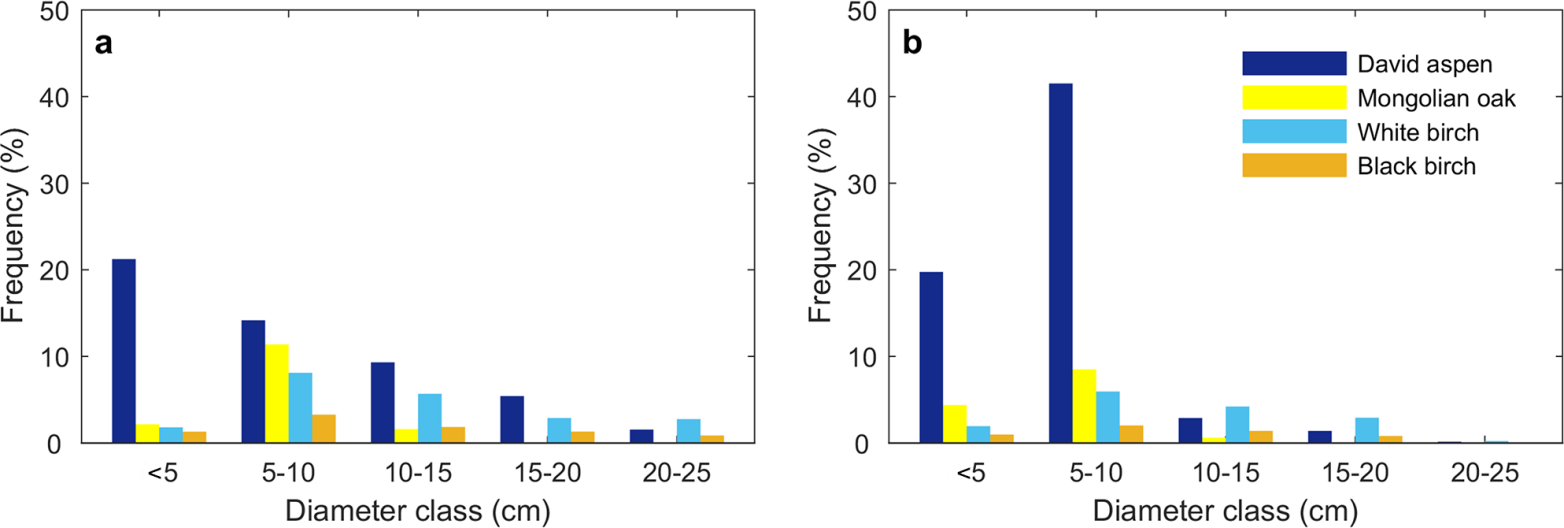
Tree stem diameter distributions of four tree species. Tree stem diameter distributions of David aspen, Mongolian oak, white birch, and black birch in (a) 2012 and (b) 2015. The total number of trees in 2012 and 2015 were 13815 and 18436, respectively.

The percentage of David aspen in 2012 decreased with increasing diameter class: most of these trees (25.9%) were in the smallest diameter class of <5 cm (Fig 3A, see Table E in S1 Table). By contrast, the highest percentage of Mongolian oak occurred in the 5–10 cm diameter class, accounting for 12.4% of the total trees (Fig 3A). Not surprisingly, similar diameter distributions were found for both white birch and black birch, with the highest percentage of these trees found in the 5–10 cm diameter class, accounting for 8.5% and 3.6% of the total trees, respectively (Fig 3A). In 2015, however, the percentage of David aspen in the <10 cm diameter class had increased to 63.1% of the total, while Mongolian oak in the <10 cm diameter class only accounted for 12.9% of the total trees (Fig 3B, see Table F in S1 Table). White birch and black birch in the 0–10 cm diameter class remained at less than 10% of the total (Fig 3B).

In all gaps released by David aspen death, the probability of self-replacement for David aspen were 94%, while the likelihoods of site loss to other species released were less than 5% (Table 1). The transition probabilities from other species to David aspen were above 50%. The major diagonal is made up of probabilities for self-replacements of the four species, with 38% for Mongolian oak, and less than 20% for white birch and black birch. The abilities of Mongolian oak replacing other species were relatively weak, with transition probability to David aspen as 4% and to white birch as 14%. The replacement probabilities of white birch and black birch were much lower, less than 10%.

**Table 1:** Transition probability matrix for mixed broad-leaved forest change.

2012	David aspen	Mongolian oak	White birch	Black birch
2015				
David aspen	94%	50%	69%	90%
Mongolian oak	4%	38%	14%	5%
White birch	2%	7%	16%	0%
Black birch	0%	5%	2%	5%

### Relationship between tree mortality and regeneration

A high frequency of mortality was found for almost all tree heights, with the highest mortality found for short trees with heights <5 m and tall trees between 10 and 15 m in 2012 (S2 Fig, see Table G in S1 Table). Surviving trees in 2015 were relatively short with a height of c. 5 m. Seedlings and saplings with heights <5 m appeared during 2012–2015; they accounted for 12.8% of all investigated trees in 2015.

**Fig 4:**
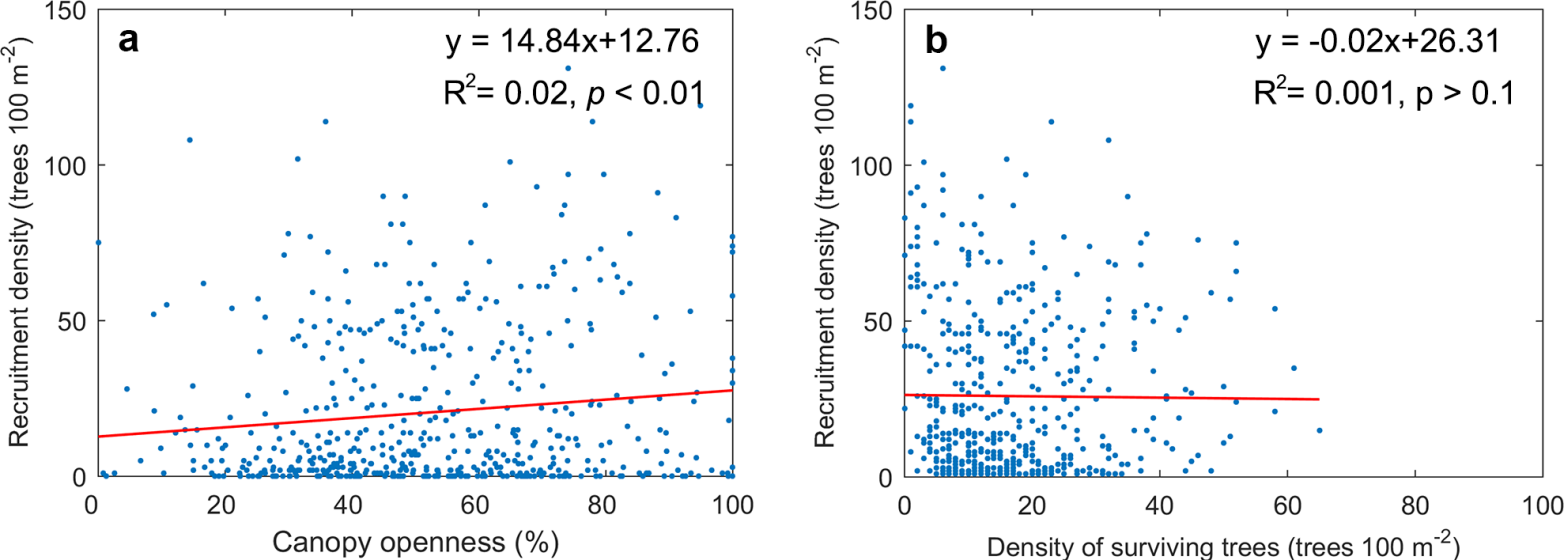
**The relationship between the recruitment density and (a) canopy openness and (b) the density of surviving trees.** Red lines show the linear relationships tested; the R^2^- and p-values indicate the fitted regression coefficients and their significance, respectively (samples size n = 600).

Recruitment density increased linearly with canopy openness (p < 0.01, Fig 4A, see Table H in S1 Table). However, no significant relationship was observed between recruitment density and the density of surviving trees (Fig 4B).

## Discussion

### Rapid regeneration of aspen after severe forest mortality

Our results rejected the hypothesis that the aspen-dominated community will succeed to a Mongolian oak-dominated community or convert to shrub and grass vegetation because of ineffective regeneration. Instead, we found abundant regeneration of David aspen following the mortality event—lacking any clear time lag. Additionally, the results revealed a growth release that was allocated towards the radial growth of young trees, which indicates a recruitment pathway for advance regeneration that existed prior to the mortality event.

The recruitment density of David aspen in this mixed forest doubled in 2015 as compared with the pre-mortality level, possibly due to the fast and effective regeneration strategy via suckering [32]. Nonetheless, we observed higher growth releases of surviving younger trees than of older ones, also indicating that David aspen regeneration was promoted. The rapid regeneration recruited the forest community and confirmed a high regrowth ability for David aspen, which is generally observed following stand-replacing disturbances, such as fire [12, 33]. Nevertheless, in this study the rapid regeneration of David aspen was associated with a different disturbance: warming-induced mortality.

The unchanged diameter distributions show that the dominant species in this forest did not shift in rank after mortality. Before tree mortality occurred in 2011–2012, both young David aspen and young Mongolian oak in the 5–10 cm diameter class were at similar proportions in the community, implying a possible invasion process of Mongolian oak; yet in 2015, the percentage of young David aspen in the 0–10 cm diameter class increased sharply and dominated the regeneration class. Meanwhile, the high probabilities of both self-replacement and replacements to other species of David aspen indicated that aspen regeneration during 2012–2015 captured most of gaps no matter what dominant species was in 2012. In contrast, the transition probabilities from David aspen to other species were relatively low. As a pioneer species in the mixed aspen-conifer forests, trembling aspen is typically replaced by shade-tolerant conifers after a canopy gap forms or partial-cutting occurs, and this conifer invasion usually prevents the recruitment of aspen [8, 14, 15]. David aspen usually occupies a similar habitat as oak and was an interim before the community developed towards oak [34–36]. However, in this study, Mongolian oak had a much lower recruitment density which even appeared to decrease after the warming-induced mortality. In addition, the transition probability from David aspen to Mongolian oak was only 4%. These results suggest that the regeneration of Mongolian oak was possibly reduced by competition with the abundant David aspen recruits for water, light, and nutrients.

### Positive relationship between mortality and regeneration

Our results suggest that warming-induced mortality might enhance regeneration. Drought stress killed the large trees (i.e., those of 10–15 m height) and it thinned the forest canopy in our study region. Recruitment density increased with canopy openness, suggesting that the asymmetric competition from crowns of surviving trees restricted forest regeneration. Warming-induced mortality possibly reduced the competition from crowns of surviving trees and thus enhanced the light availability in the forest interior and understory, which further favors the tree regeneration process. Similar effects were also observed for trembling aspen in North America for disturbances that affect the forest canopy, such as fires and heavy partial- or clear-cutting [37–40].

The enhanced forest regeneration after mortality at our site is different from that of trembling aspen in North America. Trembling aspen were recently documented to experience warming-induced mortality with damaged roots in the soil, which reduced the amounts of suckers and led to poor regeneration after mortality [5, 7, 21, 41, 42]. In addition, herbivore damage were documented to threaten aspen regeneration process in North America, by browsing new recruits after overstory mortality [43]. In the present study, however, we observed rapid regeneration after warming-induced mortality, which suggests a higher regrowth ability of David aspen in our study region than for trembling aspen in North America. This might be caused by undamaged roots. Since the study site located in a national nature reserve where grazing was forbidden, we speculated that herbivore absence was likely to prompting successful regeneration of David aspen in our study region. Although it is difficult to accurately compare the damage degrees of drought on roots between in our study region and North America, the positive effect from grazing prohibition suggested that forest managements played an important role on forest regeneration after warming-induced mortality.

### Implication for forest management in the forest-steppe ecotone

**Fig 5:**
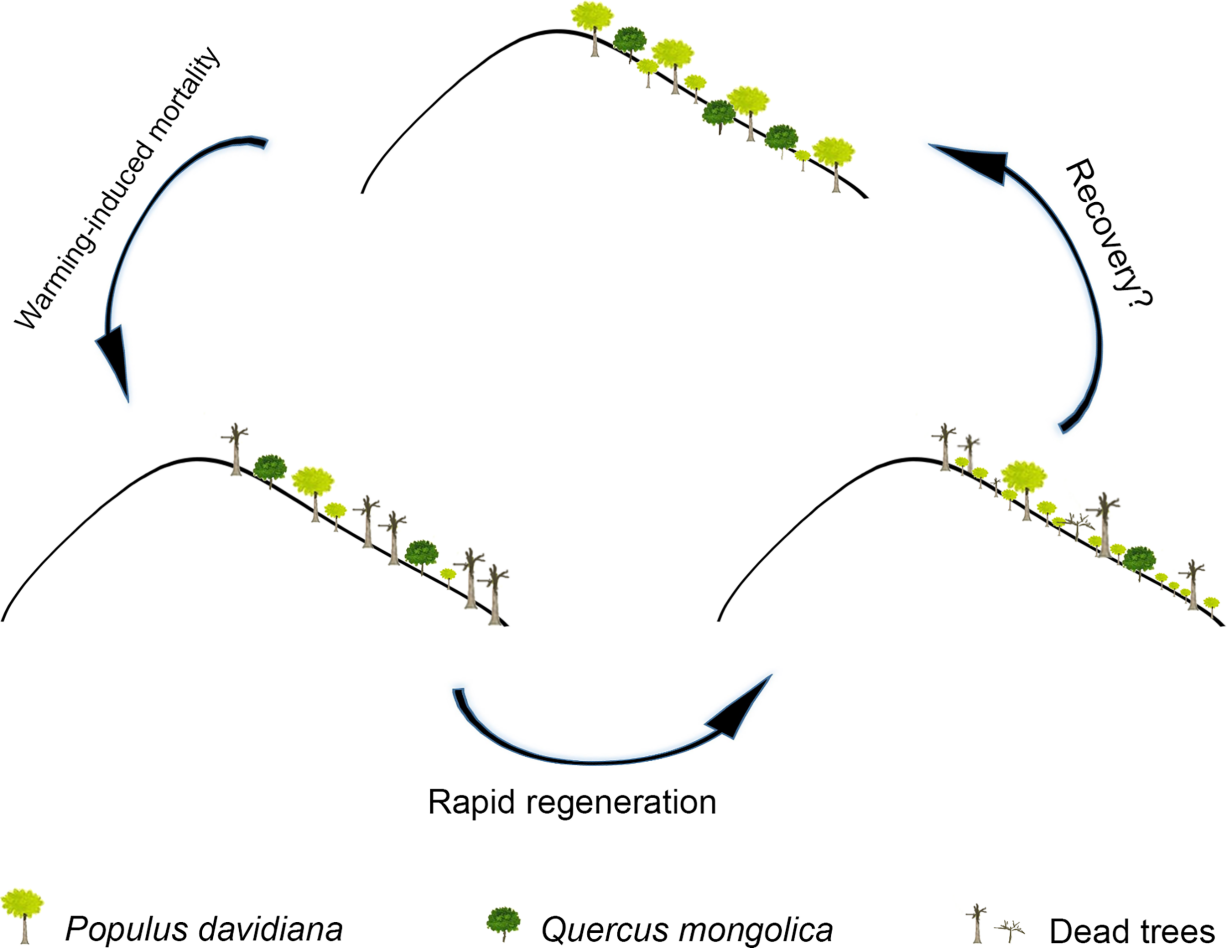
A conceptual model of the postulated recovery progress of the David aspen forest after warming-induced mortality.

How to manage the vulnerable forest in the forest-steppe ecotone remains a major challenge, while the conservation of natural forest is a national policy in China. Our results suggested a conceptual model for the recovery progress of aspen forests after mortality in this forest-steppe ecotone (Fig 5). Specifically, David aspen in semi-arid northern China has a high regrowth ability response to warming-induced mortality, as indicated by its rapid and abundant regeneration without a time lag. Tree mortality promotes aspen regeneration and increased the total number of living trees, suggesting a tendency towards compensating for losses from mortality, which was quite different from the prediction of forest decline in previous studies about forest dynamics under climate change [1, 5, 44]. Meanwhile, tree mortality also restrains Mongolian oak regeneration, indicating a regrowth process that favors aspen dominance rather than a succession to Mongolian oak dominance. Distribution of tree height indicated that dead trees were mainly saplings with height 5 m and large trees of 10–15 m height. If warming and drought continues in the future [45, 46], a high percentage of the new recruitments we observed might be killed after growing up, which may further accelerate old-aged tree replacement and rejuvenate the overall age of the forest. Meanwhile, enhanced competition from aspen saplings is likely to keep restraining regeneration of Mongolian oak, which could lengthen the succession process towards climax community. However, we only observed a relative short-term window of forest dynamics and regeneration in this study; multiple factors (e.g., drought stress and the risk of shallow rooting) may contribute to the growth of seedlings and saplings [47–49]. Whether the aspen forest recovers to its initial state requires additional work, especially over the long-term. Nevertheless, the abundant regeneration of aspen without a time lag suggests a bright future for aspen regrowth in spite of suffering severe mortality as induced by drought stress.

Our study site has a large area and covers most of the major tree species in the semi-arid region, which is rare and valuable for observing the succession process in the semiarid region of northern China. Although only containing results in a single locality, our findings provide important insights into the persistence of this vulnerable forest type in China, and possibly those facing similar threats in other parts of the world. The positive effect of mortality on regeneration was not only found in aspen, but also observed in a larch-dominated forest (*Larix sibirica*) and a birch-dominated forest (*Betula platyphylla*) in the neighboring region [50, 51]. Reduced canopy cover caused by mortality stimulated both vegetative sprouting by broadleaved trees and seed production by conifers. Although climate warming and drought threatens semi-arid forests with high rates of tree growth decline and increased tree mortality [1, 11, 52–54], semi-arid forests may be capable of a greater recruitment rate to compensate for population and community losses from mortality. Under the predicted high risk of forest loss in the future [44], we suggest that the management of forests in semi-arid regions should consider integrating the forest dynamics associated with the warming-induced mortality’s positive influence on forest regeneration.

## Conclusions

In summary, our results suggest that (1) aspen in semi-arid northern China had a high recovery ability to severe mortality, as indicated by a rapid and abundant regeneration without time lag; (2) severe mortality promoted the aspen regeneration but restrained the oak regeneration, suggesting a recovery process for aspen rather than a succession to oak; (3) intensive regeneration and serve forest mortality accelerated replacement of old-aged tree by saplings and further lengthen the persistence of David aspen; (4) forest managements (e.g. grazing prohibition) might play an important role on aspen regeneration process after warming-induced mortality.

## Supporting information

S1 Fig Tree growth increments of four species. (a) Increments of tree diameters at breast height (d.b.h); (b) Increments of tree heights at different diameter classes for David aspen, Mongolian oak, white birch, and black birch during 2012–2015.
(TIF)

S2 Fig The height distributions of living, dead, and newly recruited trees in 2015. (TIF)

S1 Table Data sets. (Table A) Increments of tree DBH. (Table B) Increments of tree height. (Table C) Density of dead trees. (Table D) Density of recruitments. (Table E) Diameter distribution of four tree species in 2012. (Table F) Diameter distribution of four tree species in 2015. (Table G) The height distributions of living, dead, and newly recruited trees in 2015. (Table H) Relationship between canopy openness, density of living trees and recruitments.
(XLSX)
