# Supplementary figures and images for: Rapid regeneration offsets losses from warming-induced tree mortality in an aspen-dominated broad-leaved forest in northern China
Source: PLoS One. 2018 Apr 6;13(4):e0195630. doi: 10.1371/journal.pone.0195630 (PMC5889172; doi:10.1371/journal.pone.0195630)

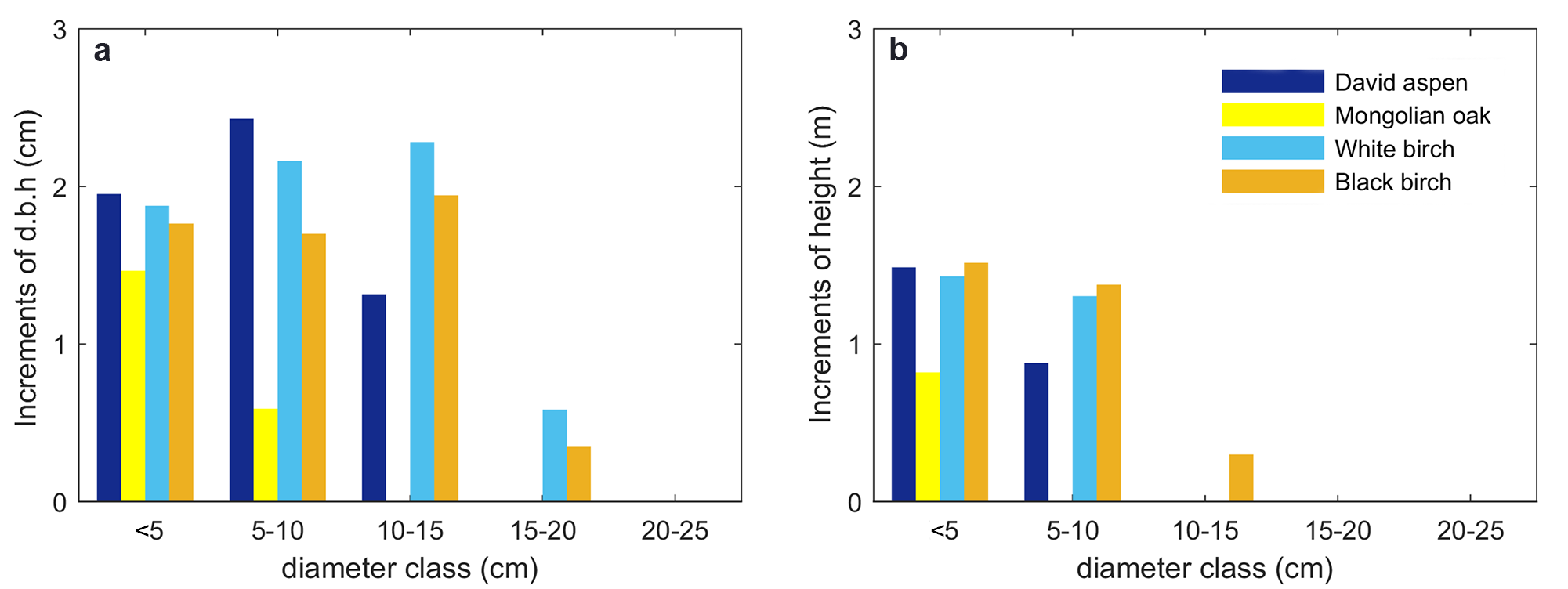

Supplement: S1 Fig — (a) Increments of tree diameters at breast height (d.b.h); (b) Increments of tree heights at different diameter classes for David aspen, Mongolian oak, white birch, and black birch during 2012–2015. (TIF) [file pone.0195630.s001.tif]

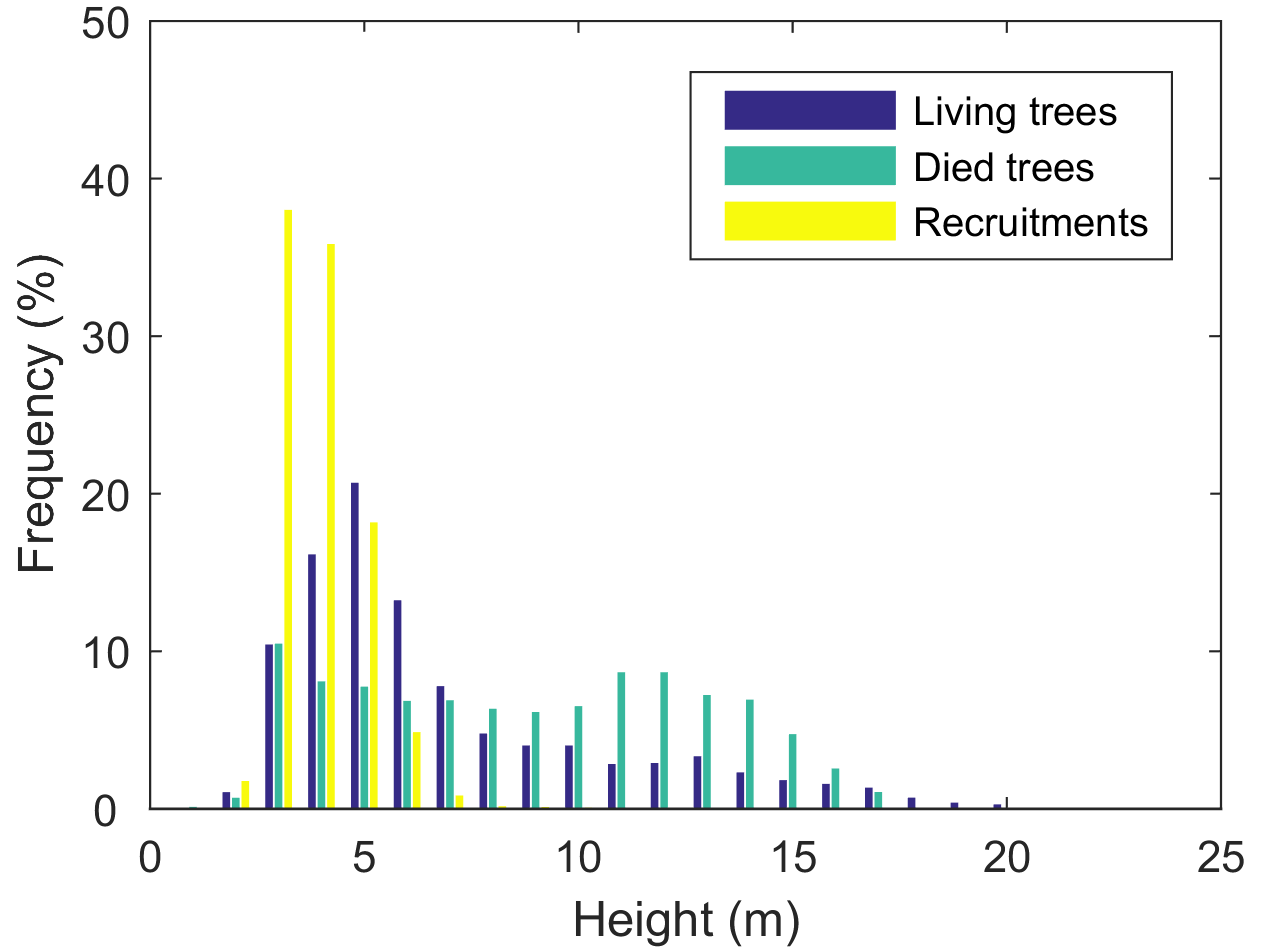

Supplement: S2 Fig — (TIF) [file pone.0195630.s002.tif]
